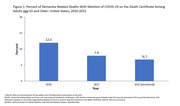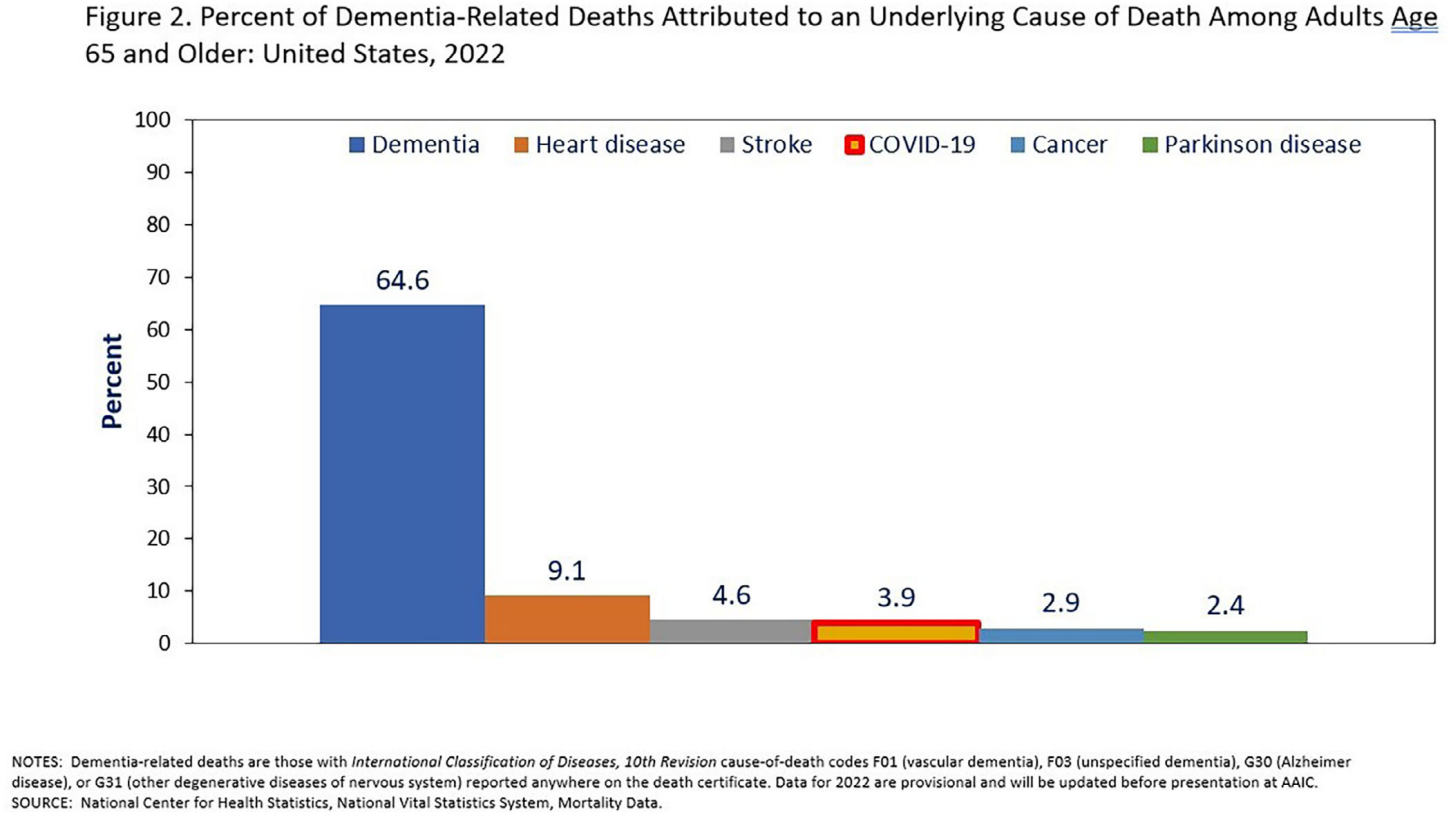# Dementia Mortality in the United States, 2020‐2022

**DOI:** 10.1002/alz.087689

**Published:** 2025-01-09

**Authors:** Ellen A. Kramarow, Betzaida Tejada‐Vera

**Affiliations:** ^1^ National Center for Health Statistics, Hyattsville, MD USA

## Abstract

**Background:**

Dementia‐related mortality increased significantly in the first year of the COVID‐19 pandemic in the United States. Explanations for the rise in dementia‐related death rates are complex and multi‐factorial. Older adults with dementia often have other chronic conditions that result in increased risk of death. Residence in institutional settings, more common among older adults and those with dementia, was associated with COVID‐19 mortality, particularly at the beginning of the pandemic. Death rates for both COVID‐19 and dementia among the older population have declined since 2020. However, dementia‐related death rates remain higher than pre‐pandemic levels. This research describes the changing contribution of COVID‐19 deaths to overall dementia mortality.

**Methods:**

This analysis uses mortality data from the 2020‐2022 National Center for Health Statistics multiple cause‐of‐death files. Dementia‐related deaths are those with International Classification of Diseases, 10th Revision cause‐of‐death codes F01 (vascular dementia), F03 (unspecified dementia), G30 (Alzheimer disease), or G31 (other degenerative diseases of nervous system) reported anywhere on the death certificate. For adults age 65 and older, we describe the underlying and contributing causes of death associated with dementia mortality by sex, age group, race and Hispanic origin, and place of death.

**Results:**

In 2022, 6.7% of death certificates for adults age 65 and older with dementia recorded anywhere on the death certificate also mentioned COVID‐19, a decline from 7.9% in 2021 and 12.0% in 2020 (Figure 1). Declines in the percentage with COVID‐19 mentioned were similar for older Black non‐Hispanic, White non‐Hispanic, and Hispanic decedents. From 2020 to 2022, the percentage of dementia‐related deaths with COVID‐19 mentioned declined for deaths in nursing homes or hospice facilities (from 13.8% to 6.8%) and increased for deaths at home (from 2.2% to 4.3%). In 2022, for all dementia‐related deaths among adults age 65 and older, the leading causes reported as underlying causes of death were dementia (64.6%), heart disease (9.1%), stroke (4.6%), COVID‐19 (3.9%), cancer (2.9%), and Parkinson disease (2.4%) (Figure 2).

**Conclusions:**

Dementia‐related mortality in the United States increased during the COVID‐19 pandemic. To date, dementia‐related death rates remain above pre‐pandemic levels, but the contribution of COVID‐19 to dementia‐related deaths has declined.